# Conjunctival sac microbiome in anophthalmic patients: Flora diversity and the impact of ocular prosthesis materials

**DOI:** 10.3389/fcimb.2023.1117673

**Published:** 2023-03-07

**Authors:** Hejia Zhao, Yanjun Chen, Yixu Zheng, Jing Xu, Chenyu Zhang, Min Fu, Ke Xiong

**Affiliations:** ^1^ Department of Ophthalmology, Nanfang Hospital, Southern Medical University, Guangzhou, Guangdong, China; ^2^ School of Public Health, Southern Medical University, Guangzhou, Guangdong, China; ^3^ Department of Ophthalmology, Zhujiang Hospital, Southern Medical University, Guangzhou, Guangdong, China

**Keywords:** ocular surface flora, microbiome, ocular prosthesis, anophthalmia, conjunctival sac

## Abstract

**Purpose:**

To explore the changes of bacterial flora in anophthalmic patients wearing ocular prosthesis (OP) and the microbiome diversity in conditions of different OP materials.

**Methods:**

A cross-sectional clinical study was conducted, involving 19 OP patients and 23 healthy subjects. Samples were collected from the upper, lower palpebral, caruncle, and fornix conjunctiva. 16S rRNA sequencing was applied to identify the bacterial flora in the samples. The eye comfort of each OP patient was determined by a questionnaire. In addition, demographics information of each participant was also collected.

**Results:**

The diversity and richness of ocular flora in OP patients were significantly higher than that in healthy subjects. The results of flora species analysis also indicated that in OP patients, pathogenic microorganisms such as *Escherichia Shigella* and *Fusobacterium* increased significantly, while the resident flora of *Lactobacillus* and *Lactococcus* decreased significantly. Within the self-comparison of OP patients, compared with Polymethyl Methacrylate (PMMA), prosthetic material of glass will lead to the increased colonization of opportunistic pathogens such as *Alcaligenes, Dermabacter* and *Spirochaetes*, while gender and age have no significant impact on ocular flora.

**Conclusions:**

The ocular flora of OP patients was significantly different from that of healthy people. Abundant colonization of pathogenic microorganisms may have an important potential relationship with eye discomfort and eye diseases of OP patients. PMMA, as an artificial eye material, demonstrated potential advantages in reducing the colonization of opportunistic pathogens.

## 1 Introduction

The loss of eyes poses huge burdensome on the living quality of anophthalmic patients (Kulkarni et al., 2018). Anophthalmic patients are distributed all over the world. In China alone, there are at least 300000 patients without eyes, and the population incidence rate can reach 0.3‰; Statistics show that in most cases, the common reason for the occurrence of anophthalmia was due to the eye enucleation of the eyeball in the treatment of severe terminal eye diseases such as trauma, tumor and congenital malformation of the eye (Saeed et al., 2006; Gupta and Padmanabhan, 2012; Yousuf et al., 2012; Ibanga et al., 2013; Zhao et al., 2013). Aphthalmia can cause a great burden on the life and work of anophthalmic patients; wearing ocular prosthesis is often the main solution, which help anophthalmic patients to repair esthetics and function, contribute social reintegration and improve their quality of life (Goiato et al., 2014; de Caxias et al., 2019; Society for Maternal-Fetal Medicine et al., 2019; Makrakis et al., 2021). The wearing of OP significantly contributes to patients’ aesthetic satisfaction. However, the wearing/implantation of OP could cause a large amount of latent adverse effects on patients, among which the most frequently-reported symptoms include the increased tears and secretion, pain, burning sensation, etc. At present, these adverse symptoms are mostly attributed to the stimulation of ocular prosthesis to the conjunctival sac, poor installation, insufficient daily care of ocular prosthesis and allergy to ocular prosthesis materials (Koch et al., 2016; Altin Ekin et al., 2020; Wang et al., 2020). To sum up, although the wearing of OP significantly contributes to patients’ aesthetic satisfaction, symptoms such as the increase of chronic secretions are often reported due to the heterogeneity of OP materials (Pine et al., 2011). OP may provide a suitable living environment for bacterial community, which might trigger the dysbiosis of ocular flora.

Over the last decades, bacterial culture has served as the mainstream approach to identify pathogenic bacteria (Christensen and Fahmy, 1974; Miller et al., 1976; López-Sánchez et al., 2001). In more recent studies, the ocular flora identified by culture methods in OP patients was characterized mainly by the presence of gram-positive bacteria including *Staphylococcus*, *Corynebacterium*, *Enterococcus*, etc. as well as the gram-negative bacteria of *Klebsiella*, *Pseudomona*s, *Stenotrophomonas*, etc (Mela et al., 2010; Toribio et al., 2019).

However, only a limited number of microbial species can be found in the flora culture, for the difference between the *in-vitro* environment and the *in-vivo* environment will also lead to unstable bacterial growth and may affect the bacterial flora. At present, bacterial identification based on 16S rRNA high-throughput sequencing reduces underlying confounding factors in bacterial culture and it has been widely used in the study of intestinal (Millar et al., 1996), vaginal (Yoshimura et al., 2011), skin (Rainer et al., 2020) and respiratory (Deng et al., 2022) microbiota.

As the changes of ocular flora in patients with ocular prosthesis and the possible adverse effects of its dominant communities remains still unclear, this study aimed to focus on the composition of ocular flora in OP patients. Therefore, the purpose of this study is to clarify the changes of ocular flora in OP patients based on 16s rRNA sequencing. Focus of: 1) the difference of ocular flora between normal subjects and OP patients; 2) Influence of gender, age, OP material and wearing time on ocular flora in OP patients. The specific goal is to identify the colonization of pathogenic and beneficial bacteria according to the dominant communities among different groups, so as to analyze the potential risk factors causing eye discomfort of OP patients. We successfully identified that 1) PMMA shows more advantages than Glass as OP materials; 2) Age and sex have no significant effect on the ocular flora of OP patients.

## 2 Method and materials

### 2.1 Research participants inclusion method

This study was registered in the Chinese clinical trial registry (ChiCTR1800016357). The study protocol was approved by the Ethics Committee of Southern Medical University and written informed consents were obtained from all subjects. The study was conducted in accordance with the Declaration of Helsinki. All 42 participants were enrolled in this study between November 2021 and April 2022, including 23 male and 19 female participants. Inclusion criteria are listed as follows: 1) The participants did not have any type of acute conjunctivitis, conjunctival cyst, orbital implant exposure, nor the usage of any scratched or ill-fitting prosthesis, also including phthisis bulbi and cosmetic scleral shells. 2) The participants had not been administrated with any antibiotics, drugs, probiotics or fiber supplements within past three months, nor received relevant treatments that may affect the homeostasis of the flora. 3) The participants did not have a history of anemia, gastrointestinal diseases, and chronic diseases. 4) The participants were not pregnant or nursing. 5) The participants did not receive eye drops (antibiotics, corticosteroids and NSAIDs) in the past 6 months. 6) No oral antibiotics or antibiotic eye drops have been used recently. 7) The participants had not used contact lenses in recent 2 months. 8) Because PMMA and Glass were two commonly used ocular prosthesis materials for OP patients at this stage, which were highly representative, so we selected OP patients who used PMMA and Glass as ocular prosthesis materials as the subjects of this study (Schellini et al., 2015).

### 2.2 Participants grouping method

All subjects who met the screening criteria were divided into ocular prosthesis group (OP) (n = 19) and control group (Con) (n = 23) according to whether the participant is an anophthalmic patient. Besides, OP patients are a small group, and sample collection is relatively difficult; and the number of OP patients who go to the hospital is not many, which makes it difficult to track and follow them up; Finally, due to the lack of data on the incidence rate of OP patients, only using the incidence rate of blind patients for sample size calculation will cause data bias. Therefore, we did not use the calculation of sample size, but collected samples of existing patients for experiment and analysis. At the same time, in order to compare the differences of ocular surface flora among ocular prosthesis patients in multiple dimensions, we further divided all participants of OP group into (1) Gender term: Male group (n = 11) and Female group (n = 8); (2) Age term: Under 25 (n = 8) and over 25 (n = 11); Since the number of anophthalmic patient is very small, and the inducements leading to anophthalmia widely occur in the adult population, so that we did not use the traditional threshold of 18 years old as the age, but used the threshold of 25 years old as the age group, which was also reflected in other research reports (Pine et al., 2017; Rokohl et al., 2018). And from our data collection, there was only one patient who was below 18 years old. (3) Material of ocular prosthesis term: Glass group (n = 5) and PMMA (polymethyl methacrylate) group (n = 14); (4) Material and wearing time terms: participants who wear glass ocular prosthesis for less than one year and more than one year are defined as Glass-down (n = 3) and Glass-up (n = 2), respectively. Similarly, participants who wear PMMA are defined as PMMA-down (n = 5) and PMMA-up (n = 8).

### 2.3 Questionnaire and information summary

All participants were requested to complete a questionnaire, which included two parts: the basic information section, the ocular prosthesis condition section and the eye discomfort section, the participants of OP group are required to fill in all sections, while the participants of Control group only need to fill in the first section. The basic information included: gender and age; the ocular prosthesis condition section included: reason for wearing the ocular prosthesis, implant, eye seat material, ocular prosthesis material, use time, and the eye discomfort section included: secretion degree, tears degree, foreign body sensation, and pain degree. The severity of eye discomfort is ranked from 0 to 4: 0), no discomfort; 1) some of the time, 2) half of the time; 3) most of the time; 4) all the time (Kulkarni et al., 2018). The total eye discomfort degree was calculated by adding up the scores above. The higher the score, the more uncomfortable the ocular prosthesis will be.

### 2.4 Sample collection and sequencing

#### 2.4.1 Sample collection

For bacterial analysis, each participant received ophthalmologic examinations at Nanfang Hospital and Zhujiang Hospital of Southern Medical University. Topical anesthesia was applied before collection. Subjects were arranged to sit in a clean room, and the ocular specimens were collected from the upper, lower palpebral, and fornix conjunctiva using one single disposable aseptic dry cotton swab containing the topical anesthetic agent from a random eye. Another one single aseptic dry cotton swab containing the topical anesthetic agent was used as a blank control. 42 samples were collected from all participants (19 OP patients and 23 Control subjects). After collection, the samples were stored at -80 °C until genome DNA extraction.

#### 2.4.2 Extraction of genome DNA

DNA was extracted using a DNA extraction kit (Mabio, Guangzhou, China) for the corresponding sample. The concentration and purity were measured using the NanoDrop One (Thermo-Fisher Scientific, MA, USA), measuring the OD value of genomic DNA solution (for the determination of concentration and purity, OD260/OD280 should be around 1.8, high indicates RNA pollution, low indicates protein pollution). In addition, we added CK bands (water control) during the DNA Extraction in order to avoid experimental bias (**Supplement data**: ‘16sRNA_Data’).

#### 2.4.3 Amplicon generation

16S rRNA/18SrRNA/ITS genes of distinct regions (e.g. Bac 16S: V3-V4/V4/V4-V5; Fug 18S: V4/V5; ITS1/ITS2; Arc 16S: V4-V5 *et. al*) were amplified using specific primer (e.g. 16S: 338F and 806R/515F and 806R/515F and 907R; 18S: 528F and 706R/817F and 1196R; ITS5-1737F and ITS2-2043R/ITS3-F and ITS4R; Arc: Arch519F and Arch915R *et. al*) with 12bp barcode. Primers were synthesized by Invitrogen (Invitrogen, Carlsbad, CA, USA). PCR reactions, containing 25 μL 2x Premix Taq (Takara Biotechnology, Dalian, China), 1 μL each primer (10 mM) and 3 μL DNA sample (20 ng/μL) in a volume of 50 µL, were amplified by thermocycling: 5min at 94°C for initialization; 30 cycles of 30 s denaturation at 94°C, 30 s annealing at 52°C, and 30 s extension at 72°C; followed by 10 min final elongation at 72°C. The PCR instrument was BioRad S1000 (Bio-Rad Laboratory, CA, USA).

#### 2.4.4 PCR products detection, pooling and purification

First of all, the length and concentration of the PCR product were detected by 1% agarose gel electrophoresis. Samples with bright main strip between (e.g. 16S V4: 290-310bp/16S V4V5: 400-450bp *et. al*) could be used for further experiments. We used 12 bp barcode specific primers to amplify all single samples, and the amplified PCR products were mixed according to the same amount of DNA. After 20-40 samples were mixed, the library index label was added to build the library. So the mixing here refers to the equal amount of mixing of the PCR products of a single sample, in preparation for the next step of building the library. PCR products were mixed in equidensity ratios according to the GeneTools Analysis Software (Version4.03.05.0, SynGene). Then, the PCR products of each sample were sequenced. Secondly, mixture PCR products were purified with E.Z.N.A. Gel Extraction Kit (Omega, USA). Each project selects the appropriate primers for amplification. Finally, when the final primer sequence was not known, it could be viewed in the mapping file of the analysis result package.

#### 2.4.5 Library preparation and sequencing

Sequencing libraries were generated using NEBNext^®^ Ultra™ II DNA Library Prep Kit for Illumina^®^ (New England Biolabs, MA, USA) following manufacturer’s recommendations and index codes were added. The library quality was assessed on the Qubit2.0Fluorometer (Thermo Fisher Scientific, MA, USA). At last, the library was sequenced on an Illumina Nova6000 platform and 250 bp paired-end reads were generated (Guangdong Magigene Biotechnology. Guangzhou, China).

### 2.5 Data analysis and result visualization

#### 2.5.1 Species annotation analysis

For each of the representative sequence, the silva (for 16S, 18S, chloroplast and mitochondria, self-organized, https://www.arb-silva.de/), Unite (for ITS, http://unite.ut.ee/index.php), RDP(for 16S, http://rdp.cme.msu.edu/index.jsp), Greengenes (for 16S, http://greengenes.lbl.gov/) database were used to annotate the taxonomic information by usearch-sintax (set the confidence threshold to default to ≥ 0.8). The taxonomy of the species annotation was divided into seven levels: Kingdom(L1), Phylum(L2), Class(L3), Order(L4), Family(L5), Genus(L6) and Species(L7).

#### 2.5.2 Species diversity, correlation and functional cluster analysis

Firstly, alpha diversity was applied in analyzing complexity of species diversity for each sample through 14 indices, including richness, chao1, shannon_2, shannon_e, shannon_10, jost, jost1, simpson, dominance, equitability, robbins, berger_parker, reads and buzas_gibson. Beta diversity analysis was used to evaluate differences of samples in species complexity through 9 algorithm, including bray_curtis, Euclidean, abund_jaccard, Canberra, chisq, chord, gower, weighted_unifrac and unweighted_unifrac by R software. The 16S ribosomal RNA (rRNA) gene sequencing has revolutionized the study of microbial communities in environments within the human body as well as other environment such soil or aquatic habitats (Cho and Blaser, 2012; Pflughoeft and Versalovic, 2012). Data analysis in such studies typically assigns 16S rRNA sequences to Operational Taxonomic Units (OTUs). There are multiple OTU clustering methods that had been proposed, in which the majority uses a threshold of 97% sequence identity (Seguritan and Rohwer, 2001; Schloss and Handelsman, 2005; Westcott and Schloss, 2017). LDA Effect Size (LEfSe) analyse was used to find the biomarker of each group based on homogeneous operational taxonomic unit (OTU)_table. At last, the abundance OTU_table was standardized by PICRUS to remove the influence of copy of 16S marker gene in the genome of species, then compared the Greengene ID corresponding to each OTU to the COG database to obtain cog family information and conduct subsequent analysis.

## 3 Result

### 3.1 16S RNA sequencing result

**Figure 1:**
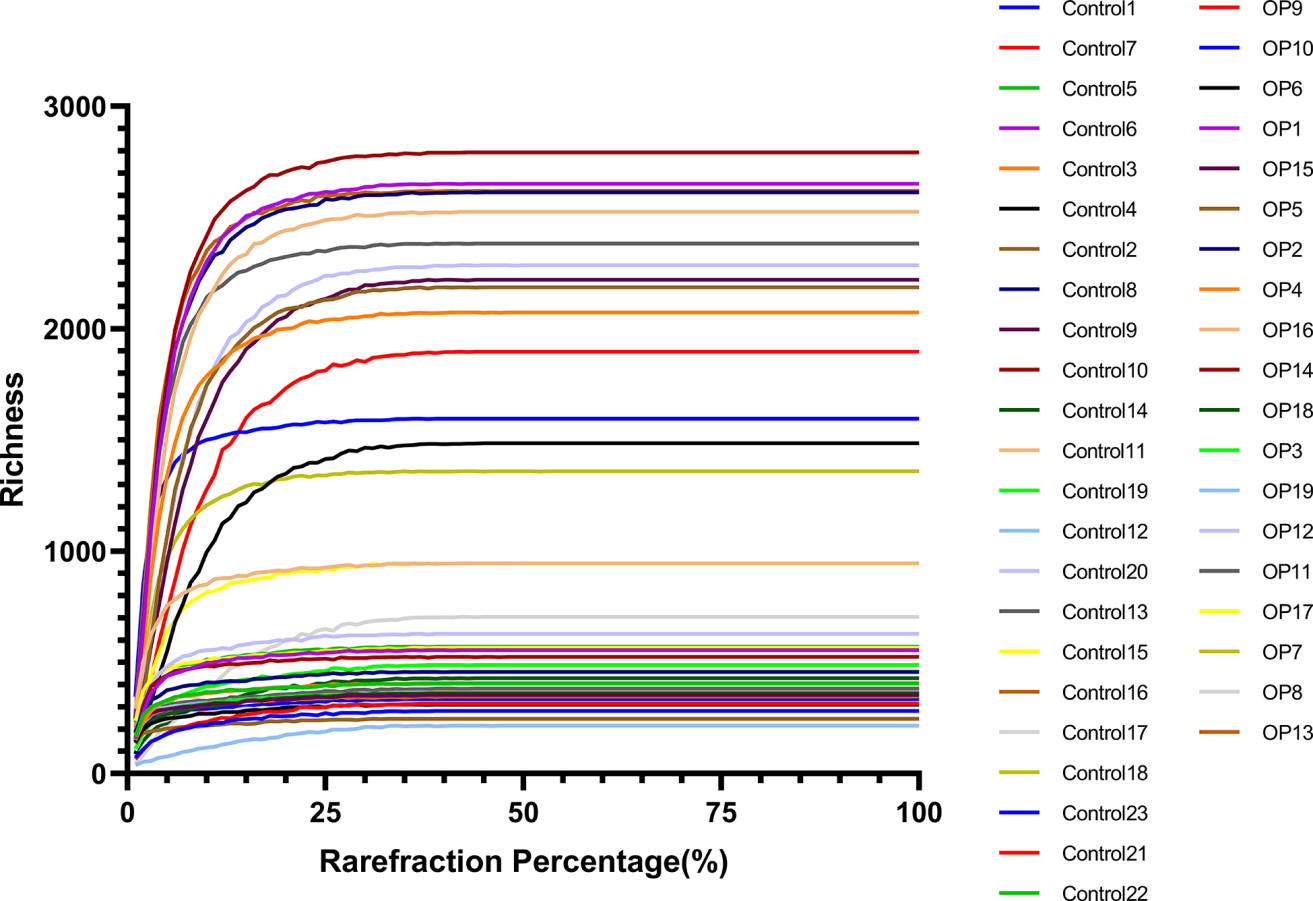
The abscissa of the raffection curve represents the proportion of the number of sequences extracted by resampling, and the ordinate represents the number of different species or diversity values.

In the process of DNA extraction, the quality of each sample was controlled: OD260/OD280 were around 1.8. Then, in the sequencing results, the range of Raw reads is 84258-92168, the range of Clean reads is 83612-91138, and the range of Clean tags is 73563-87411 Among them, when Qphred=20, the range is 99.7% - 99.9%, which is far greater than 90%. When Qphred=30, the range is 99% - 99.4%, which is far greater than 85% (**Supplement Table 1**). When the dilution curve is about 25% of the reflection percentage, the Richness of all samples has become flat (**Figure 1**). The Qphred value of the sequencing results is far beyond the qualified line. The dilution curve measures the species richness of different samples by randomly selecting a certain number of individuals from the samples, to show whether the amount of sequencing data of the sample is reasonable. Our results tend to be flat. To sum up, our sequencing depth is reasonable and can cover all bacterial groups in the sample.

### 3.2 Analysis method and participates information

In this study, the statistical analysis on the basic information of all participants showed that the difference on the history of endophthalmitis between healthy subjects and OP patients was statistically significant (P = 0.001), while the difference on other variants were not statistically significant (P > 0.05) (**Table 1**). No significant differences could be drawn in terms of gender, age and materials of ocular prosthesis among all participants in present study. Therefore, the analysis results of the included participants have a good representativeness for the population data. The statistical analysis on the clinical symptoms showed that OP patients using different OP materials had statistical significance in Secretion and Tears (P = 0.001, P = 0.005), but not in Foreign body perception and Pain (P > 0.05) (**Table 2**).

**Table 1:** Basic information statistics of participants.

Term	Control		OP		χ2	P-Value
	n	%	n	%		
Age					1.201	0.273
>25	17	73.91	11	57.89		
<25	6	26.09	8	42.11		
Sex					0.064	0.801
Male	13	56.52	10	52.63		
Female	10	43.48	9	47.27		
History of endophthalmitis					11.246	**0.001**
Yes	4	17.39	13	68.42		
No	19	82.61	6	31.58		
Systemic medication					0.987	0.321
Yes	12	52.17	7	39.84		
No	11	47.83	12	63.16		
Material					NA	NA
Glass	NA		5	26.32		
PMMA	NA		14	73.68		
Material & Wearing time					0.029	0.865
Glass-down	NA		2	10.53		
Glass-up	NA		3	15.79		
PMMA-down	NA		5	26.31		
PMMA-up	NA		9	47.37		
Cause					NA	NA
Trauma	NA		13	68.42		
Congenital malformation	NA		3	15.79		
Tumor	NA		3	15.79		

Bold values is considered statistically significant. NA: No Applicable.

**Table 2:** Clinical symptoms statistics of OP patients.

Symptom	Number of OP participants (Glass/PMMA)					Z-Score	P-Value
	0. no discomfort	1. some of the time	2. half of the time	3. most of the time	4. all the time		
Secretion	1/12	3/2	1/0	0/0	0/0	-3.210	**0.001**
Tears	0/9	2/4	2/1	1/0	0/0	-2.794	**0.005**
Foreign body sensation	3/11	1/3	1/0	0/0	0/0	-0.963	0.336
Pain	4/13	1/1	0/0	0/0	0/0	-0.783	0.434

Bold values is considered statistically significant.

### 3.3 Ocular flora analysis for control *vs* OP group

**Figure 2:**
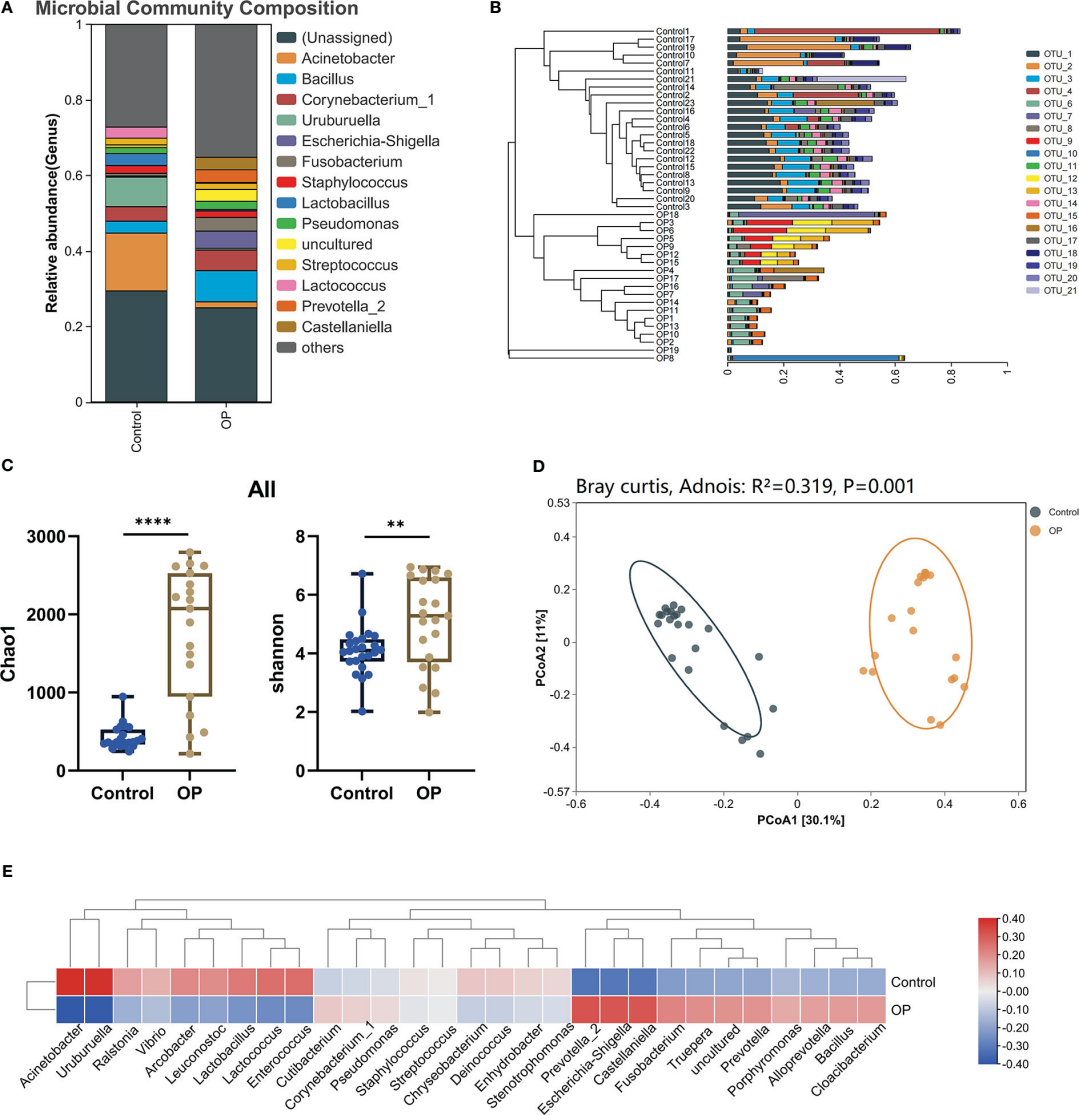
**(A)** Differences in relative mean abundances of genus in ocular microbiota between OP patients and Control subjects. **(B)** Evolution tree shows OTU level. **(C)** Alpha diversity analysis of conjunctival microbiota between OP patients and Control subjects.**(D)** Beta diversity analysis of ocular flora communities in OP patients and Control subjects visualized by PCoA plot. **(E)** The heatmap shows the dominant bacteria of OP group and Control group. **: P<0.01,****: P<0.0001.

**Figure 3:**
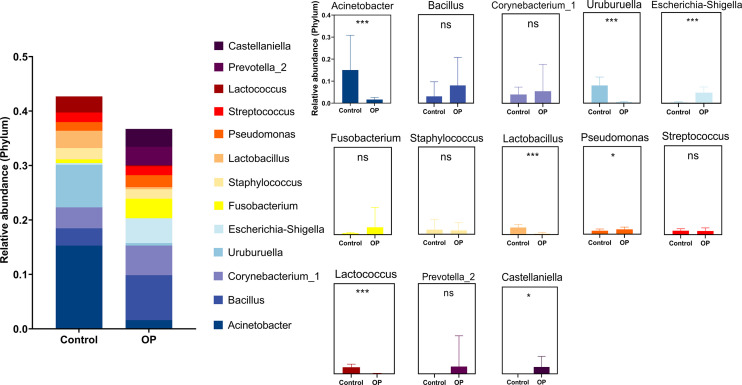
Histogram of dominate bacteria between Control group and OP group. (*: P<0.05; ***:P<0.001; ns: No Sense).

First of all, we used the stack histogram of evolutionary tree to detect the species diversity at the OTU level, and verified the superiority of sample grouping according to the clustering network (**Figure 2B**); Then, we showed the dominate flora between the control group and the OP group through stacked histograms and thermograms. In the control group, *Acinetobacter, Uruburuella, Ralstonia, Vibrio, Arcobacter, Leuconostoc, Lactobacillus, Lactococcus, Enterococcus* were the main dominant floras, and in the OP group *Prevotella_2, Escherichia Shigella, Castellaniella, Fusobacterium, Truepera, Prevotella, Porphyromonas, Alloprevotella, Bacillus; Cloacibacterium* were the main dominant flora (**Figures 2A, E**). In addition, the infiltration level of each dominant flora between Control and OP group was shown in **Figure 3**; **Supplement Table 2**. Based on the chao1 index (P<0.0001) and Shannon index (P<0.01) The alpha diversity analysis of OS microbial community in OP group was significantly higher than that in Control group, indicating that the OS microbial community in OP group was more abundant and homogeneous than that in Control group (**Figure 2C**). The beta diversity analysis results were shown in **Figure 2D**, which indicated the relationships among bacterial communities. This result indicated that the conjunctival microbiota in the Control group was significantly dissimilar to that in the OP group (R² = 0.319, P = 0.001) based on the OTU and genus profiles, which indicated the ocular flora compositions of OP patients were distinct from those of healthy subjects.

### 3.4 Ocular flora analysis for age and gender terms in OP group

**Figure 4:**
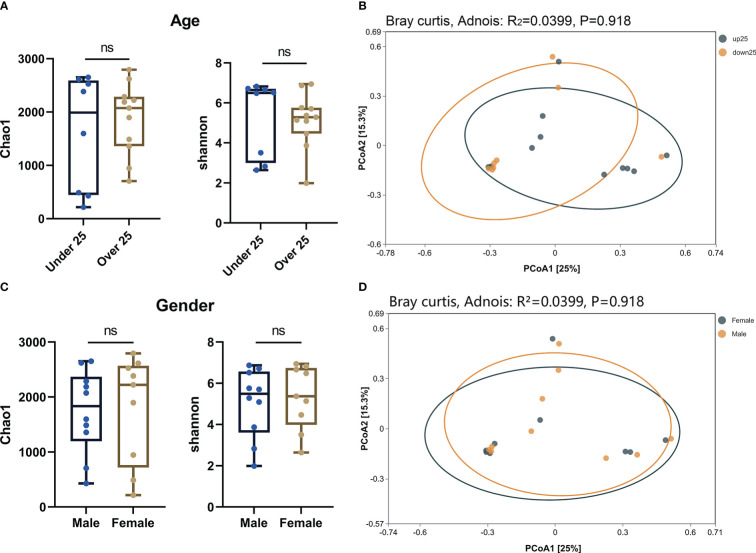
**(A)** Alpha diversity analysis of age level in OP patients. **(B)** PCoA plot shown the Beta diversity analysis of age level in OP patients. **(C)** Alpha diversity analysis of gender level in OP patients. **(D)** PCoA plot shown the Beta diversity analysis of gender level in OP patients. ns: No Sense.

In order to further explored the impact of age and gender on ocular flora in OP patients, we regrouped OP patients in terms of age and gender. Alpha (**Figures 4A, C**) and beta (**Figures 4B, D**) diversity analyses were carried out on both terms. The results showed that there were no statistical significances for alpha diversity analysis (P > 0.05) and beta diversity analysis (R² < 0.1, P > 0.05) at the age and gender levels. Therefore, age and gender of OP patients may not have significant influence on the diversity of ocular flora.

### 3.5 Ocular flora analysis for material and wearing time terms in OP group

**Figure 5:**
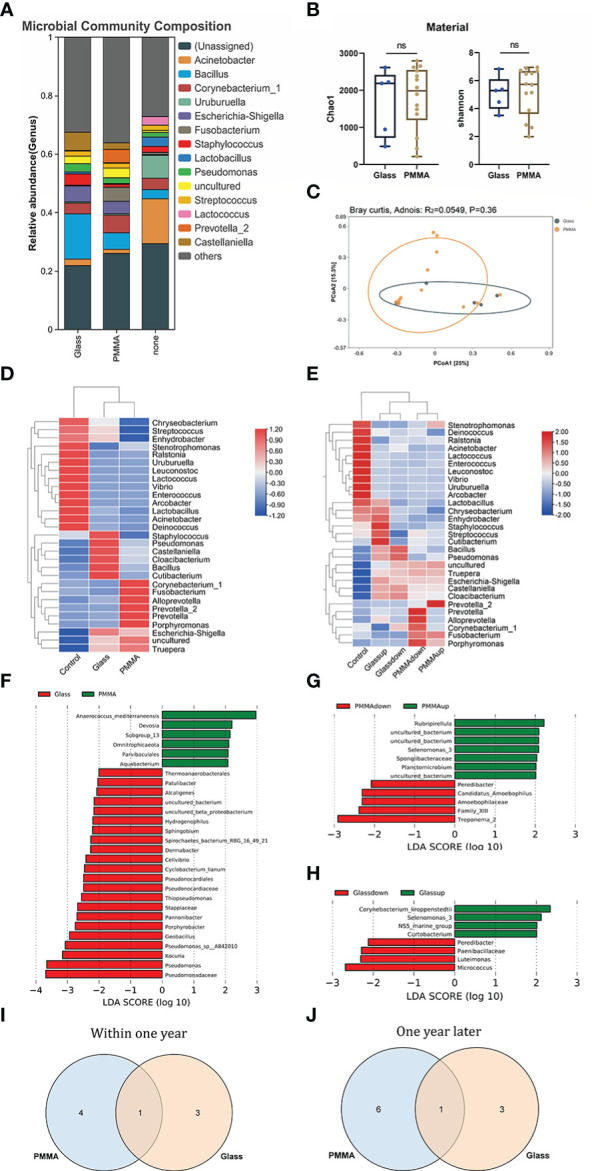
**(A)** Differences of ocular microbiota between Glass, PMMA group and Control subjects. **(B)** Alpha diversity analysis between Glass group and PMMA group. **(C)** PCoA plot shown the Beta diversity analysis between Glass group and PMMA group. **(D)** The heatmap shows the dominant bacteria of Material term. **(E)** The heatmap shows the dominant bacteria of Material & Wearing Time term. **(F)** LEfSe analysis for characteristic colony search between Glass group and PMMA group. **(G)** LEfSe analysis for characteristic colony search between PMMA-down group and PMMA-up group. **(H)** LEfSe analysis for characteristic colony search between Glass-down group and Glass -up group. **(I)** Venn plot shows the intersection colony of PMMA-down group and Glass-down group. **(J)** Venn plot shows the intersection colony of Glass-up group and PMMA-up group. ns: No Sense.

**Figure 6:**
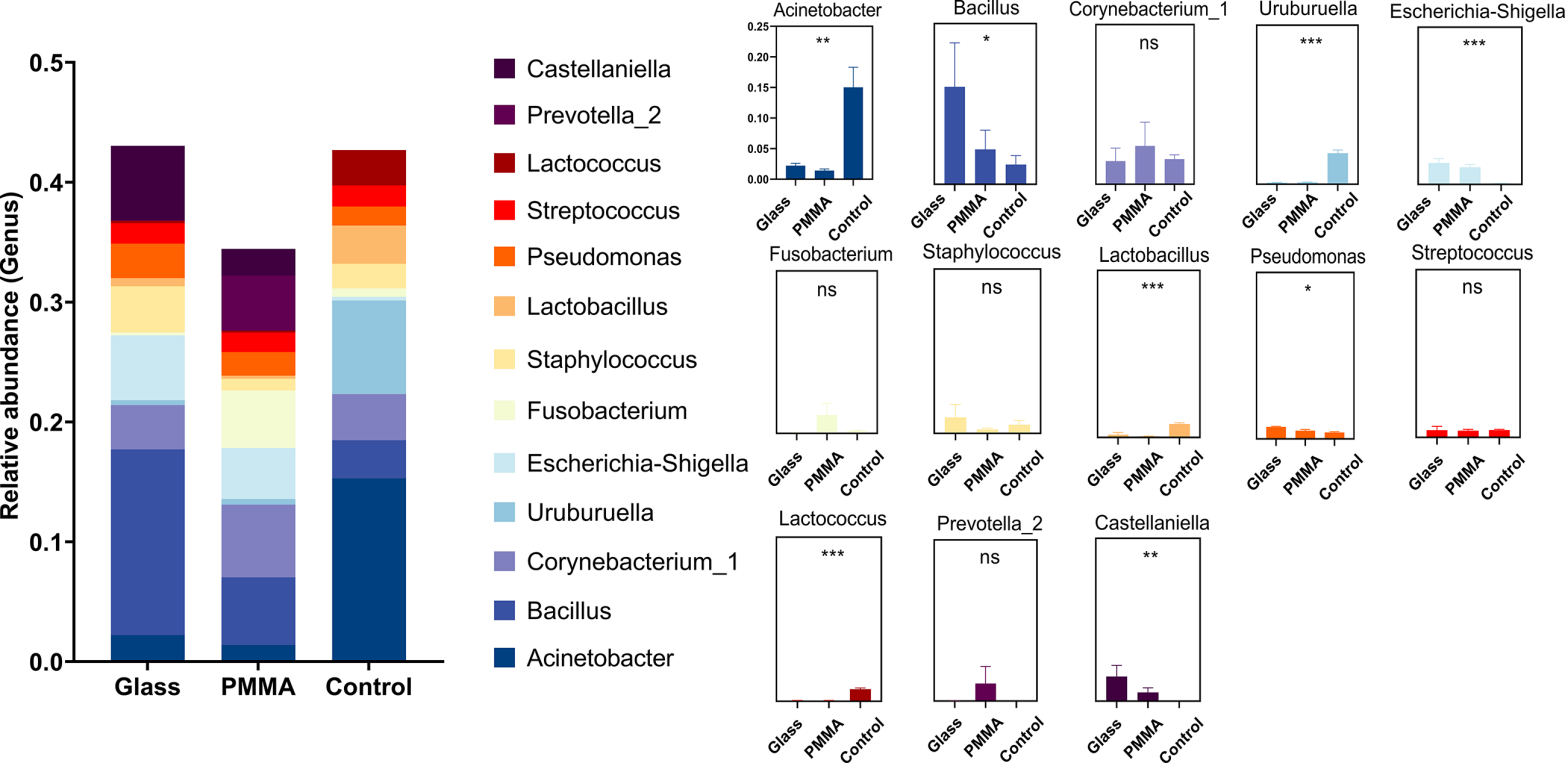
Histogram of dominate bacteria between Control group, PMMA group and Glass Group. (*: P<0.05; **: P<0.01; ***:P<0.001; ns: No Sense).

In the further exploration of the diversity in ocular flora caused by materials and wearing time, by means of diversity analysis, it had been discovered that not a statistical significance but a remarkable difference was found between respective characteristic species of each group. **Figures 5A–D** show the dominant flora of Glass group and PMMA group. And infiltration level of each dominant flora between Control group, PMMA group and Glass Group was shown in **Figure 6** and **Supplement Table 2**. After further grouping the wearing time of ocular prosthesis, **Figure 5D, E** shows the dominant flora of Glass-down group, Glass-up group, PMMA-down group and PMMA-up group. The results showed that there was no statistical significance for alpha diversity analysis (P > 0.05) and beta diversity analysis (R² = 0.0604, P = 0.292) at material and wearing time terms. To further identify the specific flora that can differentiate material and wearing time terms in OP patients, LEfSe analysis was performed (LDA score > 2.0, P < 0.05). Results showed that the abundances of *Pseudonocardiaceae, Porphyrobacter, Dermabacter, Cyclobacterium_lianum, Spirochaetes_bacterium_RBG_16_49_21, Patulibacter, Cellvibrio, Pseudomonadaceae, Alcaligenes, Pseudomonas, Kocuria, Thiopseudomonas, Sphingobium, Pseudomonas_sp:A842010, Geobacillus, Stappiaceae, Pannonibacter, Hydrogenophilus* and *Thermoanaerobacterales* were significantly higher in the Glass group than in the PMMA group, and those of *Parvibaculales, Aquabacterium, Anaerococcus_mediterraneensis, Omnitrophicaeota* and *Devosia* were lower in the Glass group than in the PMMA group (**Figure 5F**). And the abundances of *Peredibacter, Amoebophilaceae, Treponema_2* and *Candidatus_Amoebophilus* were significantly higher in the PMMA-down group than in the PMMA-up group; *Spongiibacteraceae, Planctomicrobium, Rubripirellula* and *Selenomonas_3* were lower in the PMMA-down group than in the PMMA-up group. What’s more, the abundances of *Peredibacter, Micrococcus, Paenibacillaceae* and *Luteimonas* were higher in the Glass-down group than in the Glass-up group; *Curtobacterium, Corynebacterium_kroppenstedtii, NS5_marine_group* and *Selenomonas_3* were lower in the Glass-down group than in the Glass-up group (**Figures 5G, H**). Venn plots shown the intersection of PMMA-down group and Glass-down group (**Figure 5I**) and the intersection of PMMA-up group and Glass-up group (**Figure 5J**) were taken respectively to obtain early common species (*Peredibacter*) and late common species (*Selenomonas_3*).

## 4 Discussion

Although many factors can affect the eye comfort of OP patients. However, one of the latent inducements might be attributed to the heterogeneity of exogenous OP used by patients, which easily leads to the changes of eye microecology. The disturbance of ocular flora may be another essential factor affecting the ocular comfort of OP patients. Existing studies have proved that changes in intestinal, oral, vaginal and skin microbial communities will significantly affect the homeostasis of the body (Goldsmith and Sartor, 2014; Guenin-Macé et al., 2020; Oh et al., 2021; Uehara et al., 2021). Therefore, the investigation of ocular microbiota of patients with ocular prosthesis may provide a new method to improve ocular comfortableness.

In earlier studies, bacterial culture revealed significant differences in ocular flora between healthy subjects and OP patients. The results of bacterial culture experiment showed that the richness of pathogenic microorganisms on the ocular surface of OP patients was significantly higher than that of healthy subjects, especially the increase of *Staphylococcus aureus* and *Staphylococcus epidermidis* (Guiotti et al., 2018; Altin Ekin et al., 2020). In addition, research showed that the proportion of gram-negative bacteria in patients who frequently operate and adjust prostheses will be significantly elevated (Vasquez and Linberg, 1989). However, the traditional bacterial culture method has limitations in the study of microbial communities. Some pathogens are difficult to be cultured under normal conditions, which leads to lower detection rate of bacteria compared with 16S rRNA sequencing or molecular metagenomics (Zhou et al., 2014). The emerging molecular biotechnology (16S rRNA sequencing) demonstrates a higher precision in microbial community detection. Compared with sequencing results obtained by using traditional bacterial cultures, 16S rRNA sequencing has been proven for higher sensitiveness on detecting microbial diversity (Ozkan et al., 2017).

In this study, we used high-throughput 16S rRNA sequencing to deeply identify the difference of ocular surface flora between OP patients and healthy subjects. In the comparison of ocular flora abundance between OP patients and healthy subjects, the contents of *Escherichia Shigella* and *Fusobacterium* increased significantly, while the *Lactobacillus* and *Lactococcus* decreased significantly. The existing research results show that *Escherichia Shigella* is an important ocular pathogen, which can increase patients’ susceptibility of ocular inflammation, and its increased abundance may also cause the imbalance of intestinal flora homeostasis and further affect the immune system (de Paiva et al., 2016; Huang et al., 2021). The increased of *Fusobacterium* may cause the disorder of ocular intestinal axis regulation, and lead to the occurrence and disease progression of autoimmune uveitis. Other studies showed that the infection of anaerobic bacteria may aggravate the symptoms of ocular diseases, and the detection of anaerobic bacteria may have the potential to develop into a new generation of diagnostic markers (Brook, 2001; Brook, 2008; Gunardi et al., 2021). However, compared with healthy subjects, the abundance of *Lactobacillus* and *Lactococcus* in OP patients decreased significantly. As permanent bacteria in the body, they were considered as the potential driving forces for the evolution of human immune system. As anti-inflammatory probiotics, they can also regulate the expression of Tumor Necrosis Factor - alpha (TNF - α), Interleukin – 10 (IL-10) and the infiltration level of immune cells, so as to alleviate the ocular symptoms (O'Callaghan and O'Toole, 2013; Feher et al., 2014; Yun et al., 2021). In addition, the alpha and beta diversity of OP patients was more variable than that of healthy subjects, and there was a significant statistical difference between them. The dominant ocular flora of healthy subjects was more stable than that in OP patients, indicating a change occurred in the metabolic environment. The increased in the colonization capacity of pathogenic microorganisms might be attributed to the microporous structure of ocular prosthesis materials, which may not be the suitable niche for normal bacterial species. Research have shown that the prosthesis implanted into the body for relatively longer period is easy to be colonized by pathogenic microorganisms (De Cicco et al., 1995; de Freitas et al., 2012; Veerachamy et al., 2014), which may be an important reason for eye discomfort in OP patients.

In the subsequent analysis, we determined that age and gender did not affect the diversity of ocular flora in patients with ocular prosthesis. However, alpha and beta analysis showed that age term and gender term had no effect on the ocular flora diversity of OP patients, and there was no statistical difference of them. It can’t be considered that there was a definite impact on the ocular flora between men and women in OP patients as well as the age level at present. Interestingly, in the LEfSe analysis of OP patients’ ocular prosthesis materials and use time, we found that the abundance of *Alcaligenes*, *Dermabacter*, and *Spirochaetes* in OP patients using glass as ocular prosthesis materials was higher than that in OP patients using PMMA. Current studies have shown that *Alcaligenes* is a conditional pathogen, which can cause blood flow, urinary tract, skin, soft tissue and middle ear infections (Huang, 2020; Spencer et al., 2020); *Dermabacter* has a high correlation with many diseases and has been reported to cause bacteremia in patients (Gómez-Garcés et al., 2001; Schaub et al., 2020); The colonization of *Spirochaetes* can cause uveitis, ocular syphilis and ocular leptospirosis (Rathinam, 2002; Chao et al., 2006; Duan et al., 2020). The ocular flora of OP patients using PMMA has a high abundance of *Aquabacterium* and *Devosia*. As neutral bacteria, the pathogenicity of these two bacteria remains unclear to human, but animal experiments have indicated Devosia to be protective for the mice kidneys (Zhao et al., 2016; Sun et al., 2021). According to the results of LEfSe analysis, glass as the ocular prosthesis material may have more pathogenic microorganisms colonized on its surface, while PMMA is mainly colonized by neutral bacteria, which seems to have more beneficial as an ocular prosthesis material.

Within the limitation of 16S rRNA sequencing technique, present study cannot provide the detection of fungi or viruses in the eyes of OP patients (Mahmoudi et al., 2018; Merle et al., 2018). Secondly, the enrolled number of OP patients is still insufficient and for future study, a larger-scale sample collection will be required. Although our study successfully presented a rather detailed exploration on the diversity of flora, functional prediction of flora in OP patients could be deepen. Thus, further extension of our work could be carried out on the analysis of functions and pathways of ocular flora.

In line with these findings, we can reach the following conclusions: First of all, the ocular flora species of OP patients and healthy subjects were significantly different, and the colonization of bacteria species experienced a major change. Secondly, in OP patients, age and gender terms have not been found to have significant influence on the ocular surface flora. Last but not least, compared with glass, PMMA manifested a certain degree of anti-colonized ability for pathogenic bacteria and featured as a potentially better ocular prosthesis material.

## Data availability statement

The raw data supporting the conclusions of this article will be made available by the authors, without undue reservation.

## Ethics statement

The studies involving human participants were reviewed and approved by Ethics Committee of Southern Medical University. The patients/participants provided their written informed consent to participate in this study.

## Author contributions

KX and MF conceived the study, critically reviewed the intellectual content of the manuscript and made substantive revisions to the important contents of the manuscript. HZ was the major contributor to the research and the writing of the manuscript. YC and CZ provided technical support and revised the manuscript. YZ and JX provided valuable suggestions. All authors contributed to the article and approved the submitted version.
