# Supplementary material for: Conjunctival sac microbiome in anophthalmic patients: Flora diversity and the impact of ocular prosthesis materials
Source: Front Cell Infect Microbiol. 2023 Mar 7;13:1117673. doi: 10.3389/fcimb.2023.1117673 (PMC10027910; doi:10.3389/fcimb.2023.1117673)
Supplement: Supplementary file 1 [file DataSheet_1.pdf]

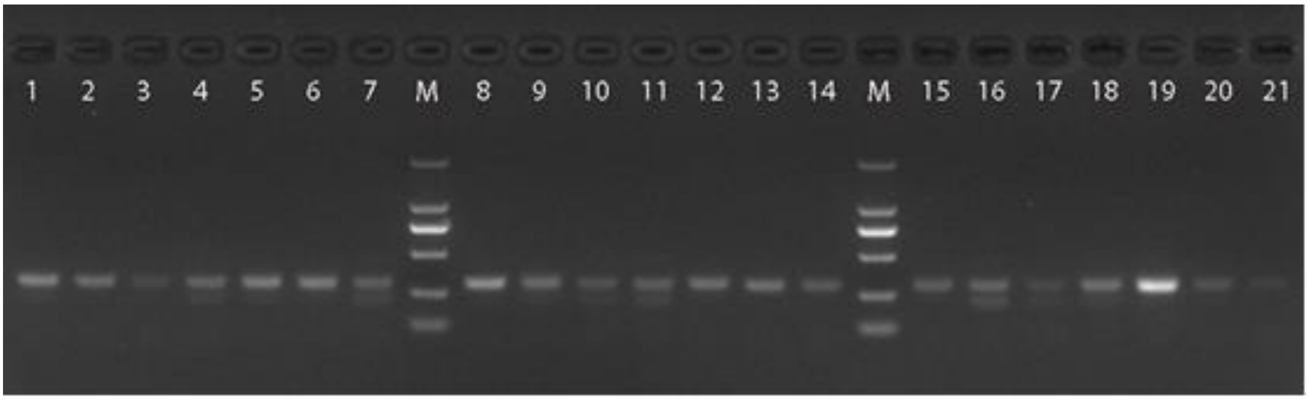

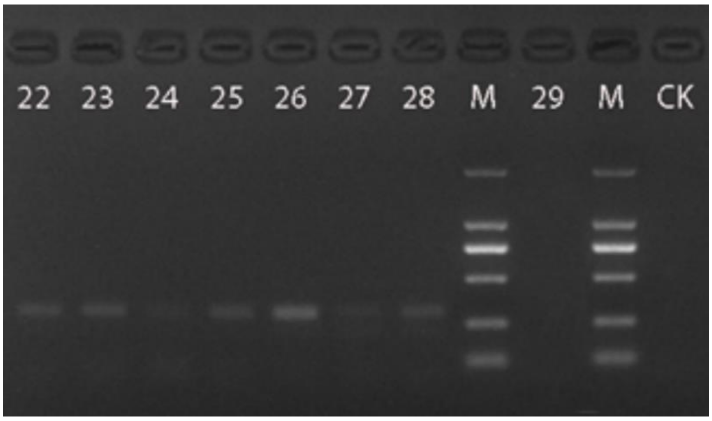

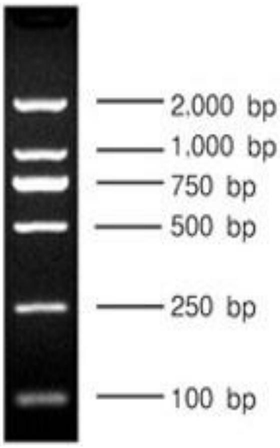

In OP Group, Blank Control results are located in band 29. 3, 17, 21, 22, 23, 24, 25, 27, 28 bands cannot be sequenced due to low concentration, so the remaining 19 samples are selected for analysis.

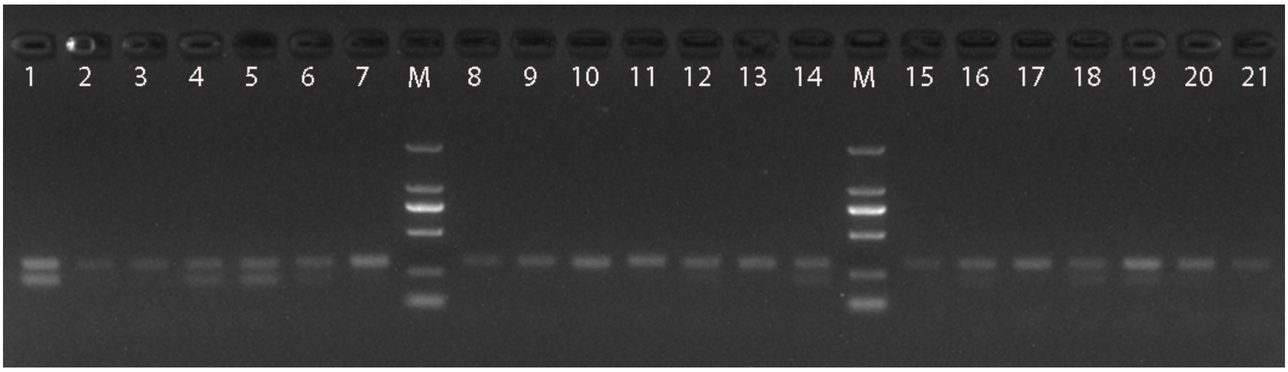

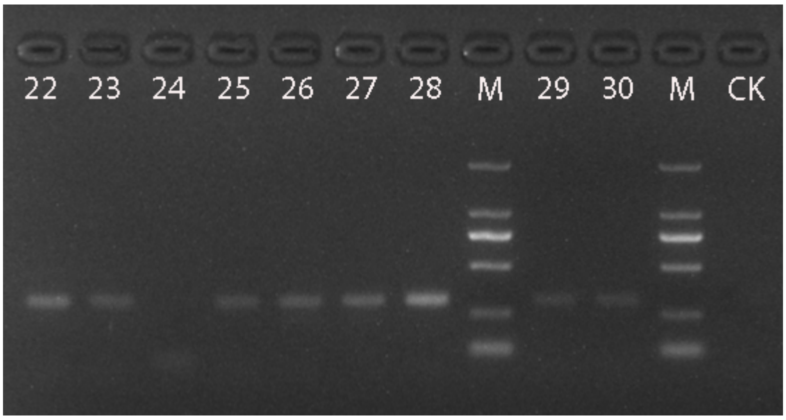

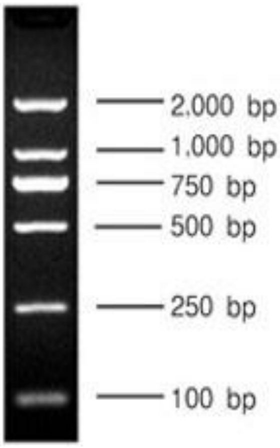

In Control Group, During the sequencing process of the control group, 2, 8, 15, 21, 24, 29 and 30 bands were too low to be sequenced, so the control group used 23 other band samples.
